# Mouse papillomavirus infection persists in mucosal tissues of an immunocompetent mouse strain and progresses to cancer

**DOI:** 10.1038/s41598-017-17089-4

**Published:** 2017-12-05

**Authors:** Nancy M. Cladel, Lynn R. Budgeon, Karla K. Balogh, Timothy K. Cooper, Sarah A. Brendle, Neil D. Christensen, Todd D. Schell, Jiafen Hu

**Affiliations:** 1 The Jake Gittlen Laboratories for Cancer Research; Pennsylvania State University College of Medicine, Hershey, Pennsylvania United States of America; 2 Department of Pathology, Pennsylvania State University College of Medicine, Hershey, Pennsylvania United States of America; 3 Department of Comparative Medicine, Pennsylvania State University College of Medicine, Hershey, Pennsylvania United States of America; 4 Department of Microbiology and Immunology, Pennsylvania State University College of Medicine, Hershey, Pennsylvania United States of America; 5 Present Address: Charles River Laboratories – Contractor Supporting: National Institute of Allergy and Infectious Diseases (NIAID) Integrated Research Facility, Division of Clinical Research, 8200 Research Plaza – Fort Detrick, Frederick, MD 21702 United States of America

## Abstract

Mouse papillomavirus has shown broad tissue tropism in nude mice. Previous studies have tested *cutaneous* infections in different immunocompromised and immunocompetent mouse strains. In the current study, we examined *mucosal* infection in several immunocompetent and immunocompromised mouse strains. Viral DNA was monitored periodically by Q-PCR of lavage samples. Immunohistochemistry and *in situ* hybridization were used to determine viral capsid protein and viral DNA respectively. All athymic nude mouse strains showed active infections at both cutaneous and mucosal sites. Interestingly, NOD/SCID mice, which have a deficiency in T, B, and NK cells, showed minimal disease at cutaneous sites but developed persistent infection at the mucosal sites including those of the anogenital region and the oral cavity. Three strains of *immunocompetent* mice supported mucosal infections. Infections of the lower genital tract in heterozygous (immunocompetent) mice of the NU/J strain progressed to high grade dysplasia and to carcinoma *in situ*. Anti-MmuPV1 neutralizing antibodies were detected in the sera of all immunocompetent animals. Our findings demonstrate that the mucosae may be the preferred sites for this virus in mice. The mouse model is expected to be a valuable model for the study of mucosal papillomavirus disease, progression, and host immune control.

## Introduction

Human papillomaviruses (HPVs) are obligate factors for the development of cervical cancer, which is responsible for the deaths of 250,000 women worldwide each year^1^. In addition, these viruses are increasingly implicated in head and neck cancers, anal cancers, and some skin cancers^2,3^. The three existing vaccines against HPVs are all prophylactic in nature and, while effective in preventing new infections of important subsets of papillomaviruses, offer little help to the many people already infected with the virus^4^. In addition, the uptake of the vaccines has been disappointingly low meaning that those people unable to clear the disease will continue to be at risk for the development of cancer over time^5^.

Papillomaviruses are species-specific and therefore it is not possible to study a HPV in an animal model. For many years, the cottontail rabbit papillomavirus (CRPV) model was the system of choice for several laboratories including our own, in part because CRPV lesions progress to cancer over time^6–10^. Much about the immunology, molecular biology and malignant potential of papillomaviruses has been learned using this system and it is anticipated that the model will continue to be a valuable resource in the years to come^11^. However, there are limitations to the model. For one thing, most HPV-associated cancers are of mucosal origin and the CRPV lesions are cutaneous^12^. In addition, reagents for the rabbit are quite limited relative to those for the most common laboratory animal, the mouse. Unfortunately, until 2011 when Ingle *et al*. reported finding Mouse papillomavirus 1(MmuPV1) in a colony of nude mice in India^13^, no mouse virus had been identified that could infect a common laboratory strain^14,15^.

The discovery of MmuPV1 intrigued the papillomavirus research community although enthusiasm was tempered by the early report that the virus was strictly cutaneous in nature^13^. A number of laboratories began to study the virus with most work assuming cutaneous tropism^16–20^. Sundberg *et al*. looked at strain and site differences and noted the formation of trichoblastomas on the dorsal skin^21^. Handisurya *et al*. also looked at strain differences and T cell involvement in clearance^17^. They showed that T cell depletion via anti-CD3 antibody rendered immunocompetent animals permissive for cutaneous infections. Wang *et al*. studied immunologic control of the virus and noted cutaneous viral persistence in one strain of immunocompetent mice, the hairless SKH-1^18^. This work was followed up by that of Jiang *et al*. in a paper in which the utility of this animal was demonstrated for the study of clearance of PV disease^19^. Uberoi *et al*. reported that systemic immunosuppression induced by a high dose of UVB promoted cancer development in MmuPV1 infected ear skin of FVB/NJ immunocompetent mice^20^.

In our laboratory, we found and reported on the first *mucosal* infections with the MmuPV1 virus and have definitively shown that oral, vaginal, anal and penile tissues are all highly susceptible to the virus, putting to rest the idea that the virus is restricted to cutaneous sites^22–25^. These observations were further confirmed by studies in another group^21,26^. In addition to the active anogenital infections and dysplasia in these animals, we have also observed that the single circumvallate papilla of the mouse tongue is uniquely susceptible to infection by the virus. This site is comparable to back of the tongue sites so commonly found in oral papillomavirus-associated cancers in humans, for which an increasing incidence is reported in younger male Caucasians^27^. We anticipate that this new mouse model will be of use in studying progression of oral papillomavirus disease.

Active infections can be readily established in immunocompromised animals at mucosal sites^22–24^. To study viral-host interactions, an immunocompetent mouse strain with intact immune response is desirable. Different immunocompetent mouse strains including C57BL/6, hairless SKH-1, and FVB/NJ have revealed differences in cutaneous site susceptibilities in previous studies^17–21,28^. We were interested in following up on these observations and in determining whether we could identify a mucosally susceptible immunocompetent strain as well. The experiments reported in this manuscript were designed to expand on our observations of mucosal MmuPV1 infections by investigating several different mouse strains. Among the animals selected, we first tested immunocompetent C57BL/6 mice and SKH-1 hairless elite mice. They showed strong immune responses to MmuPV1 infection and cleared the infection quickly. We then decided to investigate the heterozygous siblings of the homozygous immunocompromised NU/J, Hsd: NU and B6 animals, which we had previously shown to be permissive for viral infections^17,21^. These heterozygotes are immunocompetent. To follow the infections longitudinally, we monitor viral DNA copy numbers via QPCR analysis of DNA isolated from lavage samples, which we collect regularly over time^23^. This allows us to use a small number of animals to obtain robust data. It obviates the need for large numbers of animals to be sacrificed over time while still allowing for extensive data collection. The lavages have proven to be a powerful tool and have been used to study vaginal, penile, anal and oral infections^23^. We found that the NU/J, Hsd: NU and C57BL/6 *heterozygotes* were permissive for infection at the anogenital tissues. Furthermore all NU/J mice proceeded to develop carcinoma *in situ* in the vaginal infected tissues by 7.5 months post infection. Interestingly, the cutaneous tissues of these same mice showed only subclinical infections and were not permissive for papillomavirus lesion development. These important findings provide opportunities for the study of mucosal papillomavirus infections and malignancies under the influence of an intact immune system and in a biologically relevant site, the vaginal canal. They further help to cement the utility of this new MmuPV1 model for the study of papilloma diseases, progression, immunological response and viral-host interaction.

## Results

### Immunocompetent SKH1-Hrhr and C57BL/6J mice were susceptible to MmuPV1 infection at mucosal sites but cleared the infection quickly

Previous studies have demonstrated that adaptive immunity is sufficient to eliminate MmuPV1 infection in outbred hairless euthymic SKH1-Elite (Crl: SKH1-Hrhr) and C57BL/6J immunocompetent mice at cutaneous sites^19,20^. Whether or not mucosal sites, including the lower genital tract, were susceptible to MmuPV1 infection was not tested. Four SKH1-Elite and eight inbred C57BL/6J mice were infected vaginally with MmuPV1 (Table 1).

**Table 1:** The mouse strains used in the current study.

Mouse strain	Genotype	Deficiency	Immune treatment	Infected Sites	
				Cutaneous	Mucosal
SKH1-Elite	Wild type	Normal	N/A		Vaginal tract
C57BL/6 J	Wild type	Normal	N/A		Vaginal tract
		Normal	N/A		Oral cavity
C57BL/6 J	Wild type	Normal	CD4 and CD8 T cell depletion		Anal canal
NU/J heterozygotes	Foxn1^nu/+^	Normal	N/A	Tail, muzzle	Vaginal, anal and oral
Hsd: NU heterozygotes	Foxn1^nu/+^	Normal	N/A	Tail, muzzle	Vaginal, anal and oral
C57BL/6 heterozygotes	Foxn1^nu/+^	Normal	N/A	Tail, muzzle	Vaginal, anal and oral
NOD/SCID		T, B, and NK	N/A	Tail, muzzle	Vaginal, anal and oral
Hsd: NU homozygotes	Foxn1^nu/nu^	T	N/A	Tail, muzzle	Vaginal, anal and oral
NU/J homozygotes	Foxn1^nu/nu^	T	N/A	Tail, muzzle	Vaginal, anal and oral
IFNα/βR^−/−^	IFNα/βR^−/−^	Type I IFN receptor	N/A	Tail, muzzle	Vaginal, anal and oral

Infection was followed by the detection of viral DNA in vaginal lavage, a tool that has proven to be very robust in our hands^23^. Viral DNA was detected in the lavages at week two post-infection in both strains but was undetectable at week four post-infection (Supplementary Fig. 1A and B). We detected anti-viral antibodies in serum samples from these infected animals (Supplementary Fig. 1C and D) indicating that transient mucosal infections probably occurred^26^. Serum conversion was also reported in cutaneously- infected immunocompetent mice^19^. These findings suggest that both immunocompetent mouse strains are susceptible to MmuPV1 infection at vaginal mucosae, and that the infections rapidly clear.

Eight C57LB/6J mice were used to test the ability of these immunocompetent mice to sustain oral infection. Viral DNA was detected in the DNA from oral lavages by QPCR at week three post-infection in six of the mice and became undetectable after week four (Supplementary Fig. 2A). Two mice with the highest viral DNA copy numbers were sacrificed for histological analysis. In neither of these animals was viral DNA detected by *in situ* hybridization. Serum samples from all animals were harvested for antibody detection at week seven post-infection. All eight orally-infected B6 mice generated detectable antibodies against the mouse papillomavirus (Supplementary Fig. 2B). No viral DNA was detected at the infected sites by *in situ* hybridization analysis in any of the animals at week seven post infection, supporting the rapid clearance of disease.

In our previous studies of immunocompromised mice, we have found anal sites to be somewhat less permissive to MmuPV1 infection than vaginal and oral sites^23^. Previous studies have shown that CD4 and CD8 T cells are crucial to the elimination of MmuPV1 in immunocompetent mice at cutaneous sites^17,18^. We, therefore, decided to deplete CD4 and CD8 cells in three C57BL/6J mice by using anti-mouse CD4 (Clone GK1.5) and anti-mouse CD8 (clone 2.43 against CD8a) for seven weeks following anal viral infections. The CD4 (Supplementary Fig. 3A) and CD8 (Supplementary Fig. 3B) levels were evaluated after the termination of the experiment by one-color flow cytometry analysis^29^. Although significantly lower levels of CD8 were found in the depleted animals (Supplementary Fig. 3C, P < 0.05, unpaired Student T-test), the CD4 T cell population was not significantly different between the two groups (Supplementary Fig. 3C, P > 0.05, unpaired Student T-test). Anal infections were detected at week five post-infection (Supplementary Fig. 3D and E, P < 0.05, unpaired Student T-test) but no viral DNA was detected after week six post-infection. We concluded that the observation that MmuPV1 infection could not be sustained in the anal mucosae of immunocompetent C57BL/6J mice, even under antibody depletion conditions, may have been due to insufficient CD4 T cell depletion.

### NOD/SCID mice were susceptible to mucosal infections but resistant to cutaneous infections

**Figure 1:**
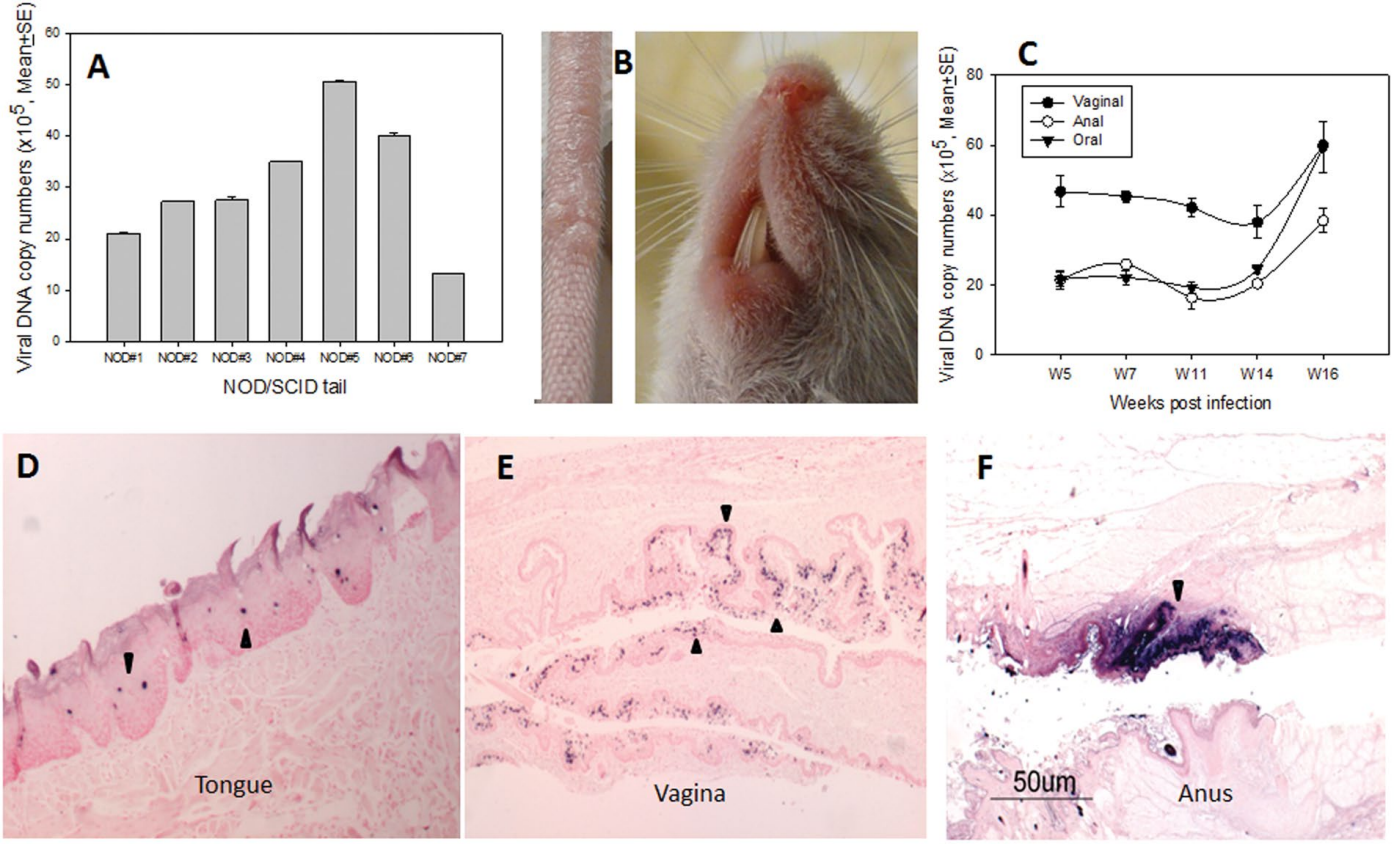
Viral infections were found in NOD/SCID mice at cutaneous and mucosal sites. Viral DNA was detected in infected tail tissues (**A**) even though only one visible lesion was detected on one of the eight tails, and no visible muzzle lesion was found (**B**). Viral DNA was tracked at mucosal sites by measuring DNA in vaginal, anal, and oral lavage samples by Q-PCR (**C**). Viral DNA was further confirmed at the dorsal tongue (**D**, 10×, arrows), the vaginal tract (**E**, 10×, arrows) and the anus (**F**, 10×, arrows) by *in situ* hybridization.

A previous study reported that NOD/SCID mice were resistant to cutaneous MmuPV1 infections (19). In the current study, we tested eight NOD/SCID mice for both cutaneous and mucosal infection. Although no visible cutaneous lesions were detected in seven of the eight infected mice, we were able to detect viral DNA at ten weeks post-infection in the infected tail tissues of all animals by Q-PCR (Fig. 1A). One small tail lesion on one of the infected mice was observed; no muzzle lesions were detected (Fig. 1B). On the other hand, high levels of viral DNA were detected in vaginal, anal, and oral samples of all animals by Q-PCR (Fig. 1C). Selected oral (Fig. 1D), anal (Fig. 1E), and vaginal (Fig. 1F) sites were subjected to *in situ* hybridization following sacrifice and all were positive (arrows). These observations have confirmed that the mucosal tissues of another immunocompromised mouse are permissive for MmuPV1 infection but that the cutaneous sites are refractive for productive viral infection.

### Persistent infection was established in heterozygotes at mucosal sites

**Figure 2:**
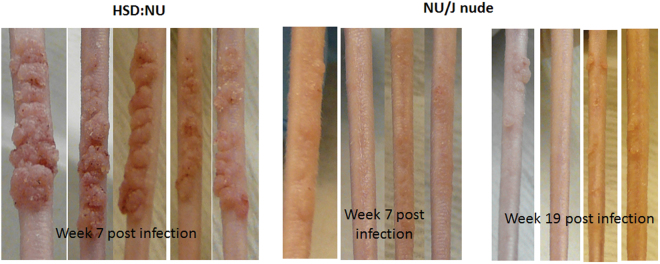
Tail infections in athymic HSD: NU mice were more vigorous than those in athymic NU/J mice. Hsd: NU (N = 5) and NU/J (N = 4) mice were infected on the tails with MmuPV1. Lesions became visible around week two post-infection in both mouse strains. However, lesions on Hsd: NU mice (Left) persisted and became significantly larger than those on NU/J nude mice by week seven post-infection (Right). The lesions on NU/J nude mice remained small and did not grow to significant size by week nineteen post-infection. One of the four NU/J nude mice did not develop any lesion at the tail site. Similar results were found at the muzzle sites in these two immunodeficient mouse strains.

A previous study reported that inbred nude mice of the NU/J strain were resistant to viral infection at back sites (19). We tested both the immunocompromised homozygous NU/J (nude) and immunocompetent heterozygous NU/J (hairy) mice of this inbred strain in the current study. We also tested the HSD: NU outbred (homozygous) nudes, which have been used in our previous studies. Animals were infected at both mucosal and cutaneous sites. Lesions were detected at the tail and the muzzle sites of the inbred NU/J nude mice although the lesions were significantly smaller when compared with those in the outbred nude mice (HSD: NU) (Fig. 2).

**Figure 3:**
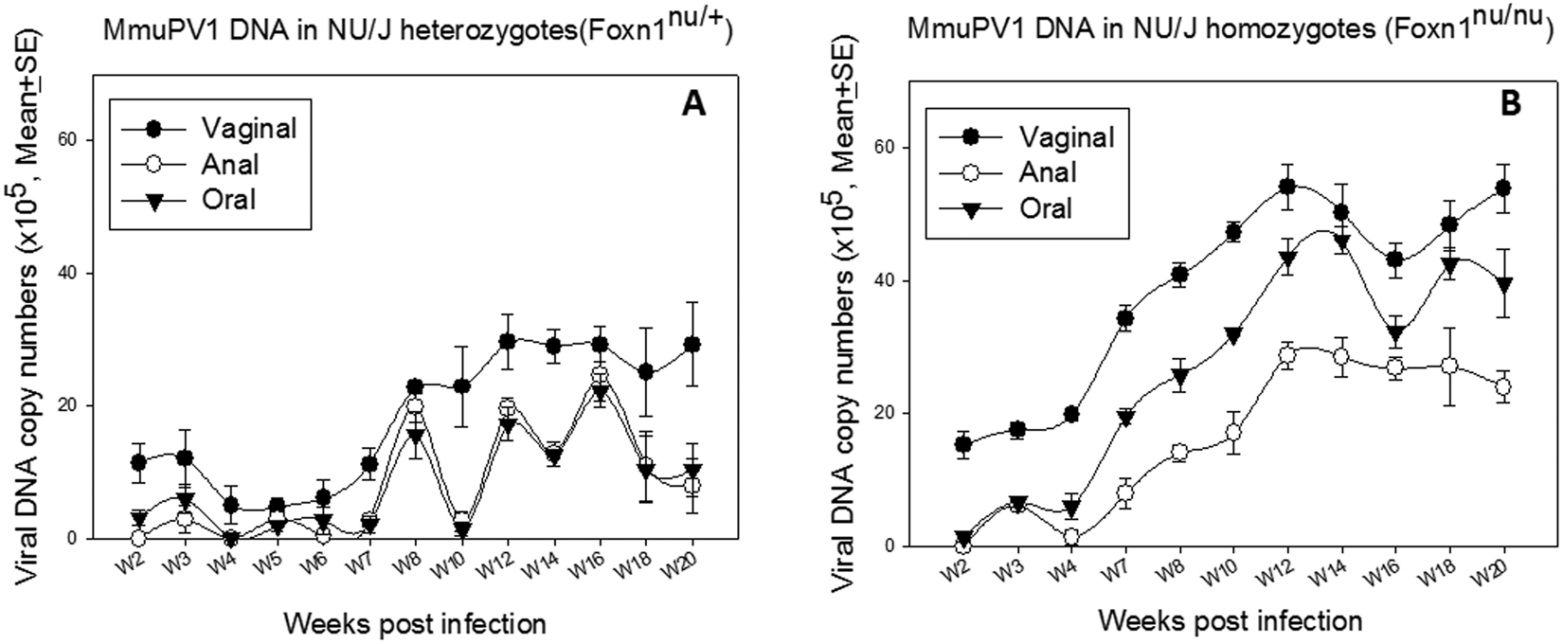
Persistent mucosal infection was established in the *immunocompetent* NU/J *heterozygous* mice. Four homozygous inbred NU/J nude mice (**A**) and four inbred heterozygotes (**B**) were tested for infection at mucosal sites. Viral DNA was monitored periodically over the infection course at the mucosal sites (the vagina, the anus and the oral cavity). Both strains supported persistent infections. Significantly higher viral DNA copy numbers were detected in the homozygous nude mice (P < 0.001, after week seven post infection, unpaired student T test).

On the other hand, comparable *mucosal* infections were detected in both strains of nude mice. The viral DNA copy number in oral, anal and vaginal lavages was monitored through week 20 post viral infection in all three strains. Infection was detected in all although significantly lower levels of viral DNA were seen in the heterozygous mice when compared with the corresponding homozygous mice (Fig. 3A and B, P < 0.001 after week seven post infection, unpaired Student T-test).

**Figure 4:**
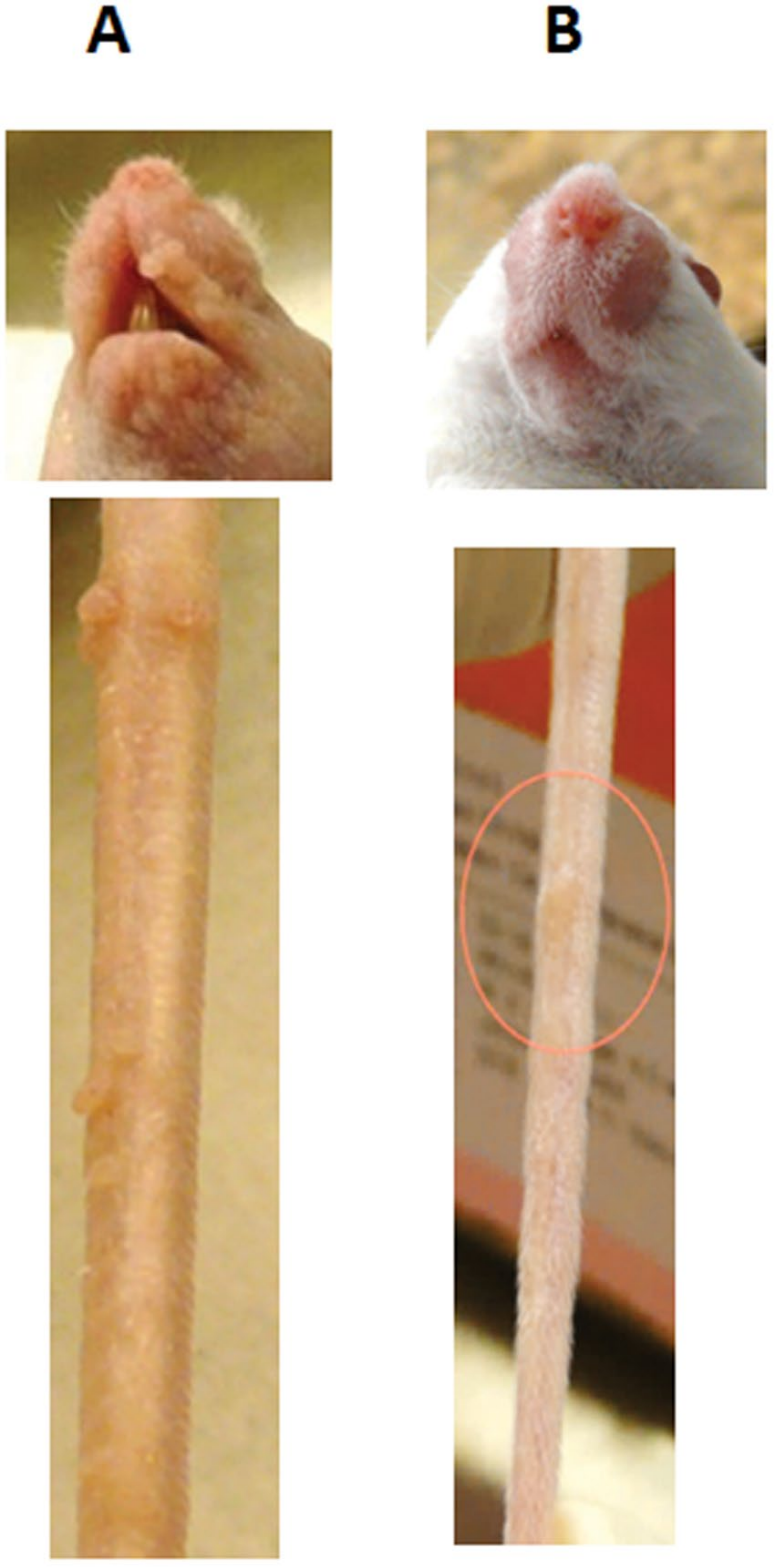
NU/J homozygotes and heterozygotes displayed differential response to infections at cutaneous sites. Four homozygous inbred NU/J nude mice and four heterozygous inbred hairy NU/J mice were tested for infection at cutaneous sites (tail and muzzle). Lesions were detected at most tail and muzzle sites in the homozygotes (a typical lesion shown in (**A**), but only a single tail lesion was observed in one of four heterozygotes and that lesion later regressed (**B**). No muzzle lesions were observed in the heterozygotes.

The viral infection in all animals persisted over time. Unlike homozygous mice with visible lesions at both muzzle and tail sites (Fig. 4A), no muzzle lesions were detected in the heterozygous animals and only, a single lesion was detected on one of the tails of these animals; this lesion regressed within two weeks (Fig. 4B).

### Advanced disease was found in MmuPV1-infected NU/J heterozygotes

**Figure 5:**
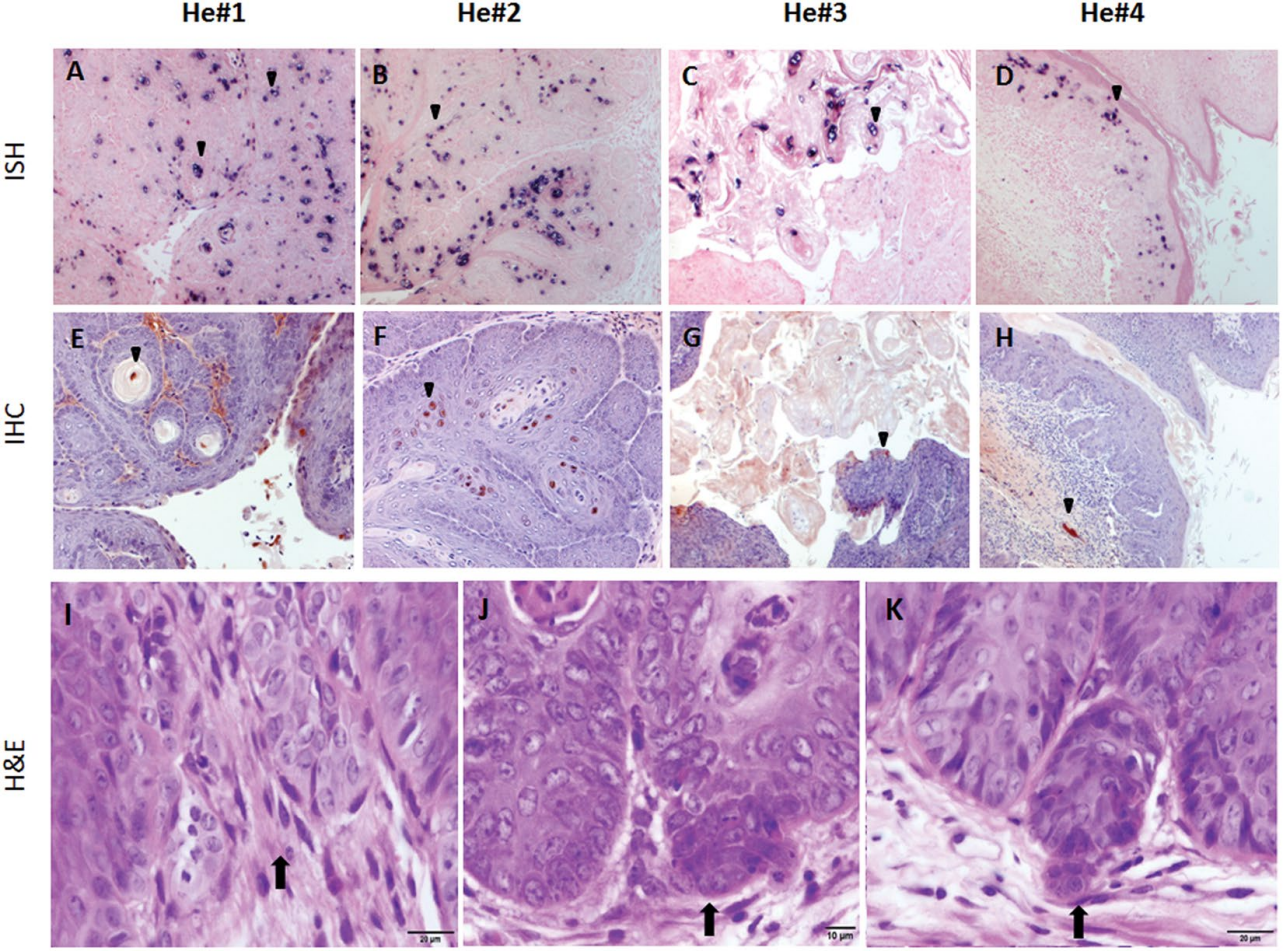
NU/J heterozygous (immunocompetent) mice developed productive viral infections in vaginal tissues; carcinoma *in situ* was detected at 7.5 months post infection. MmuPV1 DNA was detected in infected vaginal tissues in NU/J heterozygous mice (He#1-#4) upon sacrifice at 7.5 months post-infection by *in situ* hybridization (ISH, **A**–**D**, 10×, and arrows). Capsid protein was detected by immunohistochemistry using an in-house anti-MmuPV1 monoclonal antibody (MPV.B9, (**E**–**H**), 10×, and arrows). Cell morphology was examined by **H** and **E**. Carcinoma *in situ* was diagnosed in the vaginal tracts of all animals and ambiguous stromal micro-invasion by basal cells (**I**–**K**, 20×, and arrows) and scattered foci of ambiguous micro invasion were found in some

Four NU/J heterozygous (He#1-He#4) NU/J mice were sacrificed to examine histological changes at the lower genital tract at 7.5 months post-infection. All of these animals were positive for viral DNA by *in situ* hybridization (ISH, Fig. 5A–D, 10×, and arrows) and for viral capsid protein by immunohistochemistry (IHC, Fig. 5E–H). Detailed pathological analysis revealed carcinoma *in situ* in the vaginal tracts of all four animals (Fig. 5I–K, and arrows). Ambiguous stromal microinvasion by basal cells was seen in one of the four genital tracts (H&E, Fig. 5I). Cell invasion was detected at multiple lesions (H&E, Fig. 5J–K, 20×, and arrows). No significant signals were detected in the oral cavity and the anal canal of these mice.

### MmuPV1 disease persisted in mucosal tissues in homozygous NU/J mice

**Figure 6:**
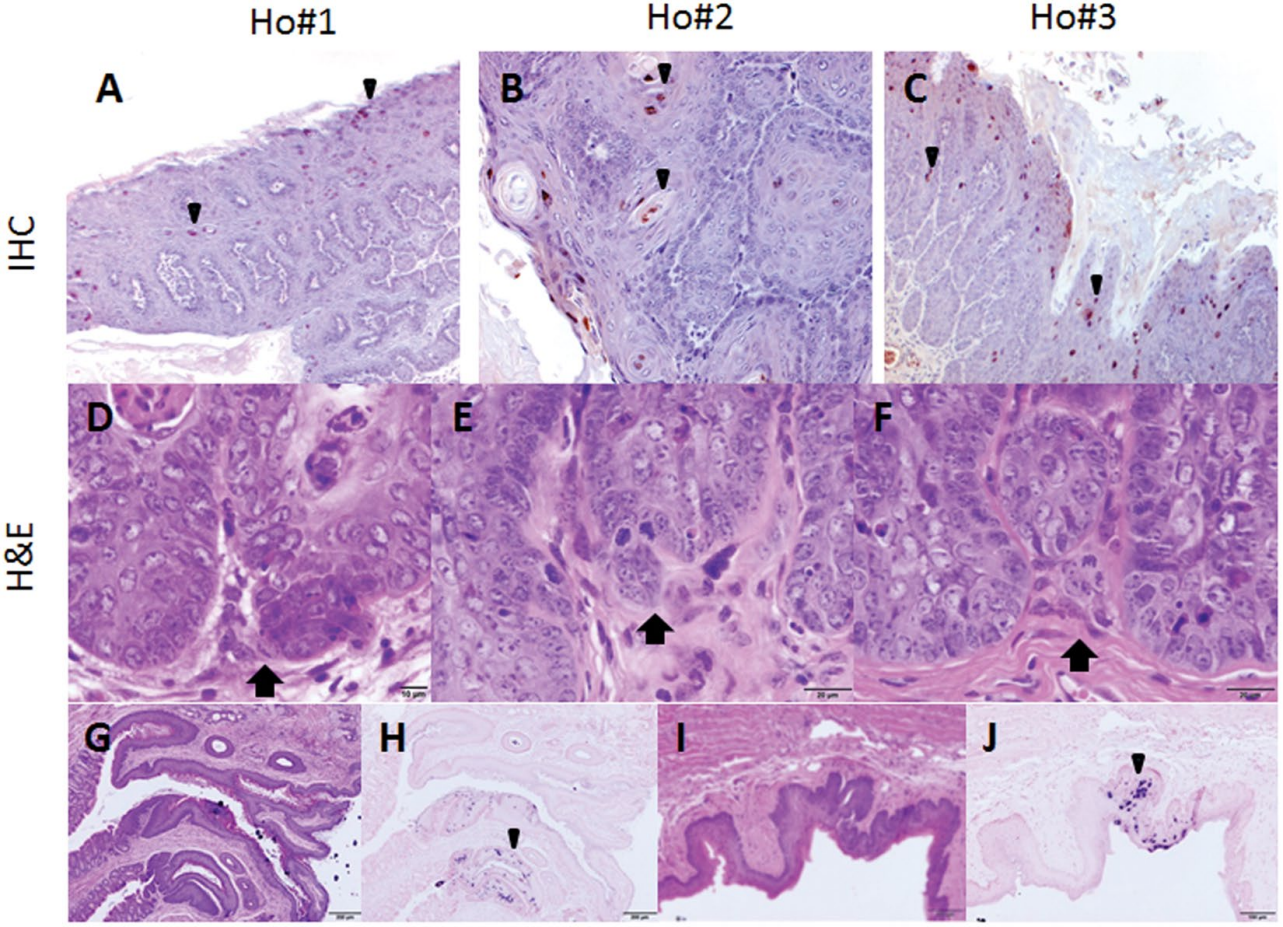
NU/J homozygous (immunocompromised) mice exhibited infections in mucosal tissues. MmuPV1-infected vaginal tissues exhibited dysplasia in the homozygous NU/J mice. Mice were sacrificed at 7.5 months post-infection and tissues were examined for histological changes. Viral capsid protein was detected in these tissues by immunohistochemistry with an in-house anti-MmuPV1 antibody (MPV.B9) (Ho#1-#3, **A**–**C**, 10×, and arrows). Scattered foci of ambiguous micro invasion were found in the lower genital tract of these homozygous NU/J mice (**D**–**F**, 20×, and arrows). Infections at the anal and oral sites were examined by histology. There was diffuse mild hyperplasia and hyperkeratosis of the stratified squamous cornifying epithelium of the anal canal (**G**, 10×, and arrows). There was minimal atypia or obvious viral cytopathic effect (CPE), with low to moderate numbers of ISH positive cells, mostly located near the recto-anal junction (**H**, 10×, and arrows). A small plaque was found on the ventral surface of the tongue just rostral to the duct of the sublingual salivary gland (**I**) and was positive for viral DNA (**J**, 10×, and arrows).

One of the four infected homozygous NU/J mice died before the termination of the experiment. The remaining three animals (Ho#1-#3) were positive for viral capsid protein by immunohistochemistry of the lower genital tract (Fig. 6A–C, 10×, and arrows). There were scattered foci of ambiguous microinvasion in these infected tissues (Fig. 6D–F, 20×, and arrows). The stratified squamous cornifying epithelium of the anal canal displayed diffuse mild hyperplasia and hyperkeratosis (Fig. 6G, 10×, and arrows). There was minimal atypia or obvious viral cytopathic effect (CPE) in the anal canal and low to moderate numbers of ISH-positive cells (Fig. 6H, 10×, and arrows), mostly located near the recto-anal junction. A small plaque was found on the ventral surface of the tongue just rostral to the duct of the sublingual salivary gland and was positive for viral DNA (Fig. 6I–J, 10×, and arrows).

### Heterozygotes of Hsd: NU and C57BL/6 (B6) nude mice also supported mucosal infections

**Figure 7:**
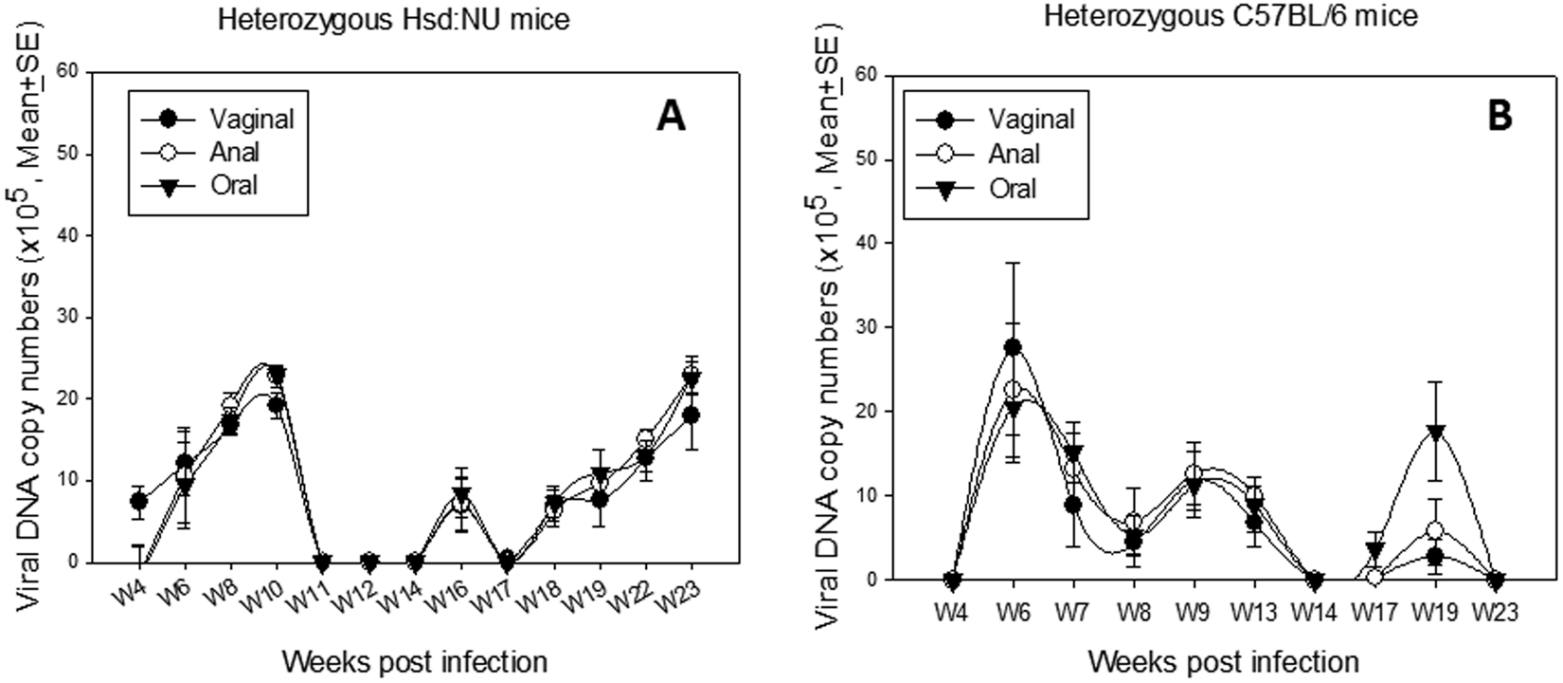
Immunocompetent heterozygous Hsd: NU and C57BL/6 mice maintain vaginal, anal, and oral mucosal infections over time. Infections in additional immunocompetent heterozygous strains of mice (Hsd: NU and B6) were also tracked over time in an experiment that is still ongoing. Heterozygous Hsd: NU mice showed persistent infection over the time courses (**A**) similar as shown in herterozygous NU/J mice (Fig. 3A). Hsd: NU heterozygotes have maintained infections up to the 23 week time point (**A**). B6 heterozygous animals (**B**) were permissive to MmuPV1 infection but showed no detectible infections at any of the sites after week 23 time point.

We had shown that immunocompetent NU/J heterozygotes not only supported mucosal MmuPV1 infections but that these infections progressed to cancer (Fig. 5). To determine if other immunocompetent strains of mice might also be permissive five Hsd: NU heterozygotes and four C57BL/6 nude heterozygotes (Foxn1^nu/+^) were infected at oral, anal and vaginal sites. Weekly or biweekly lavages demonstrated that infection was maintained in the Hsd: NU heterozygotes up to at least week 23 (Fig. 7A). The B6 Nude heterozygotes were permissive (Fig. 7B) but did not show positivity after week 23. These experiments are on-going and final results will be reported at a later date.

### Infiltration of neutrophils in the lower genital tract was detected in infected animals

**Figure 8:**
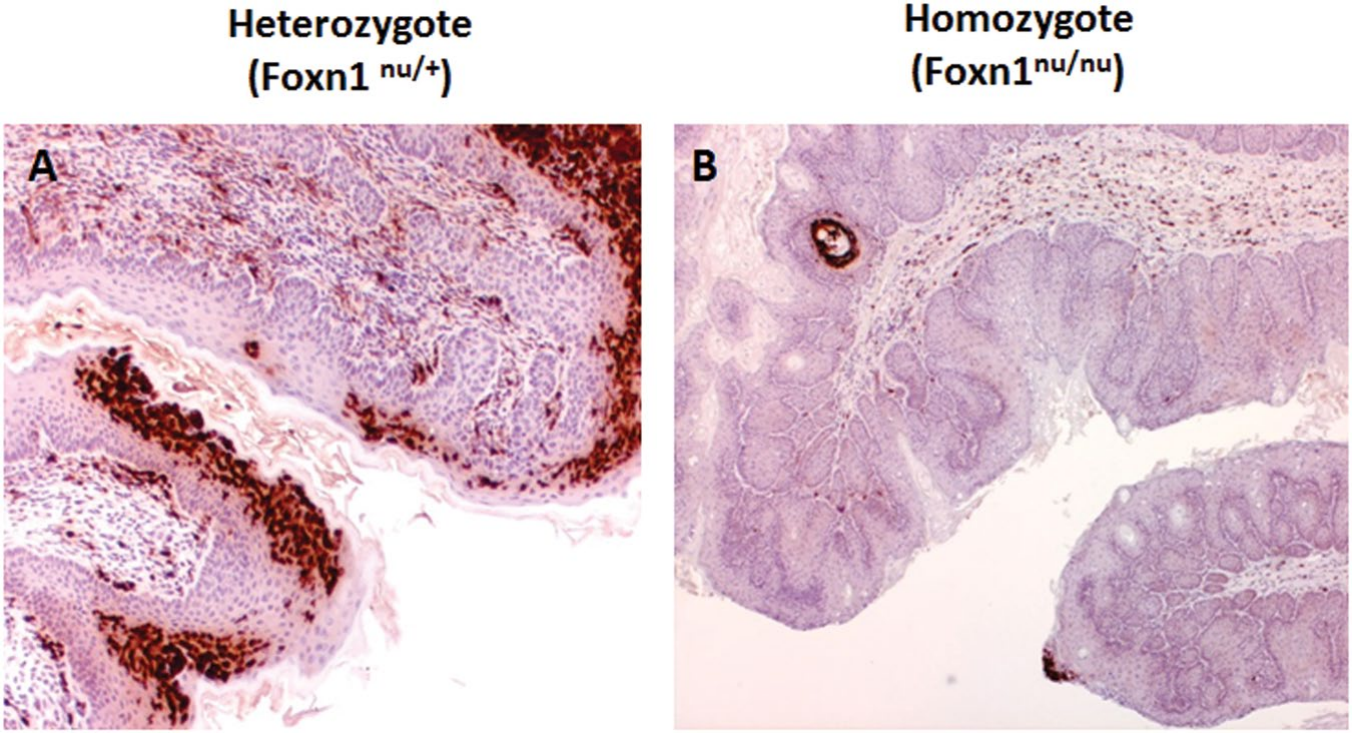
Neutrophil infiltration was more abundant in heterozygous NU/J animals than in homozygous animals. Neutrophils were detected at infected vaginal tissues in both immunocompetent NU/J heterozygous and immunocompromised homozygous NU/J mice by immunohistochemistry using anti-mouse LY6.B2 (MCA771GT, Bio-Rad). Interestingly, more neutrophil infiltration was detected in the heterozygous mice (**A**, 20×, in red) when compared to that in the homozygous mice as determined by staining (**B**, 20×, in red)

The infected vaginal tissues from all heterozygous and homozygous NU/J mice were positive for viral DNA by *in situ* hybridization (Figs 5, 6). To explore whether innate immune cells such as neutrophils infiltrated into the infected sites, we used a neutrophil-specific monoclonal antibody (LY6.B2) to detect these cells in the lower genital tract of both heterozygous (Fig. 8A) and homozygous (Fig. 8B) mice. Significantly more neutrophils were identified at the infected vaginal tissues of heterozygous mice when compared to those of homozygous mice (Fig. 8A,B).

### Neutralizing antibodies were generated in the infected animals

The sera of all heterozygous mice and two of the four homozygous mice were positive for anti-MmuPV1 antibody (Supplementary Fig. 4). These sera were able to neutralize the MmuPV1 infection of a mouse keratinocyte cell line.

### Regression of mucosal MmuPV1 infection is delayed in IFNα/βR knockout mice

To begin to look at the role of Type I interferons in innate immune control of MmuPV1 infection, IFNα/βR knockout mice (two females 2–7 L, R and one male 1–7 L) were infected mucosally and disease was tracked by quantitation of viral DNA in lavage samples^30^. Relative to annually-infected B6 mice under transient CD4 and CD8 T cell depletion (Supplementary Fig. 3) and vaginally infected wild type B6 (Supplementary Fig. 4A), disease persisted at the anal and vaginal canals for a longer time in Type I interferons receptor knockout animals (Supplementary Fig. 5A and B respectively).

## Discussion

MmuPV1 was discovered in a nude mouse colony in 2011^13^. Later, a variant was reported in the house mouse from another laboratory^31^. Most studies to date have been based on the MmuPV1 isolate from nude mice^17,18,20,21,25^. Our laboratory has done pioneering work with this virus and has demonstrated secondary mucosal infection in mice that were originally infected at cutaneous sites^25,30^. This finding demonstrates a broad tropism of this virus in athymic mice^22–24,32^. Mucosal infection was later reported in two other studies thus confirming our findings^21,26^.

Several laboratories have attempted to establish cutaneous infections in immunocompetent animals^17–21^. Handisurya, *et al*. found that Cyclosporin A treatment is required for induction and maintenance of MmuPV1-induced papillomas in immunocompetent mice^17^. Uberoi *et al*. found that several mouse strains including FVB/NJ, C57BL/6, and BALB/c mice were susceptible to MmuPV1 infection after exposure to high doses of UVB^20^. UVB radiation is associated with systemic immune suppression as measured by inhibition of DTH responses in these mice^20^. In tissues supporting MmuPV1 infection in FVB/NJ mice, persistent papillomas developed at focal regions consistent with squamous cell carcinoma, including invasion extending into follicular structures deep within the dermis^20^. Jiang *et al*., in a recent study, also observed that 20% of hairless SKH-1 mice established persistent infections^19^. These studies suggest that specific strains of immunocompetent animals are susceptible to viral infection at cutaneous sites. Most are able to eliminate the disease due to strong host immune responses but persistence and even cancers are possible^17,18^.

*Mucosal* infections in immunocompetent animals have not been reported. Our current study was designed to examine whether such infections could be established and maintained. This was of special interest to us because of the association between papillomavirus disease and cervical, penile, and head and neck cancers. In our current study, we tracked mucosal infections in several immunocompetent mouse strains by monitoring viral DNA copy numbers in lavage samples as well as by antibody generation^19^. Hairless SKH-1 and B6 mice showed transient infections at anogenital mucosae and the oral cavity (Supplementary Fig. 1). Anti-papillomavirus antibody was detected in these animals. These observations indicate that the virus is presented to the immune system even in the absence of overt disease. The antibody detected in these immunocompetent animals may be generated either from the original virus exposure or be a consequence of a latent infection that was detected following immunosuppression^19,20^. Transient infection parallels most HPV infections in the human population. Almost all people will contract HPV infections in their lifetimes, but most will clear the infections unless their immune responses are compromised^33^.

CD4 and CD8 T cells are the important components of adaptive immunity for the control of viral infections^34^. Previous studies demonstrated that depletion of either CD4 or CD8 T cells was not sufficient to enable MmuPV1 to produce lesions at cutaneous sites such as the tail of B6 mice^17–19^. Interestingly, CD4 depleted animals showed higher viral transcripts at the infected sites when compared with CD8 depleted animals indicating CD4 T cells might have helped to eradicate the infection in these animals by producing neutralizing antibodies to block the viral spread. On the basis of these observations, we simultaneously depleted both CD4 and CD8 T cells in the anally infected B6 mice and tracked viral DNA in the anal lavage. We chose to do this procedure in experiments with anal infections because it has been our observation, in immunocompromised mice, that anal infections are not as robust as oral and genital infections and we wanted to optimize opportunities for infection^23^. When we examined the CD4 and CD8 T cell populations in these mice, the depletion of CD8 T cells was more successful than that of CD4 T cells. The normal CD4/CD8 cell ratio is 2:1. We used the same amount of monoclonal antibody for each depletion. One out of the three mice failed to show an effective CD4 depletion which made the statistical analysis not significant (Supplementary Fig. 3A–C). Viral DNA peaked at week five post-infection and disappeared around week six post-infection (Supplementary Fig. 3D,E). In agreement with previous studies^17–19^, we speculate that the residual CD4 T cells may have helped to eradicate the infection in these animals by producing neutralizing antibodies to block the viral spread.

E6-specific MmuPV1 CD8^+^ T cells can eliminate MmuPV1-induced papillomas in athymic mice by adoptive transfer^18,19^. Intriguingly, we observed that NOD/SCID mice, a mouse strain that is deficient in T, B and NK cells, still managed to control cutaneous infections at subclinical levels but showed persistent infection at the three tested mucosal sites (Fig. 1). We hypothesize that host defense factors independent of and in addition to CD4 and CD8 T cells may play a key role in local viral clearance in these animals.

Innate immunity has been reported to play a role in the control of viral infection at mucosal sites^35,36^. In a previous study, IFNα/βR- mice were found to be free of MmuPV1-induced cutaneous lesions ^18^implying that this receptor may not be important for viral control. Whether latent infections were established at those sites were unclear. When we tested MmuPV1 mucosal infection in IFNα/βR- mice, a prolonged time to regression was found at mucosal sites of these mice^30^; Infections were detected up to three months post infection (Supplementary Fig. 5). These findings suggest that type I interferon might play a role in disease outcome.

Innate immune cells including NK cells and neutrophils are important in host defense against viral infections^37^. In the current study, we detected more neutrophils in the tissues of heterozygous NU/J mice with a milder disease than in those of homozygous mice with more severe disease indicating that neutrophils may have contributed to the disease outcome (Fig. 8). The role of these and other immune cells in MmuPV1 infection and persistence needs to be further investigated.

Interestingly, two immunocompetent mouse strains, the heterozygous NU/J mouse and the heterozygous Hsd: NU mouse, were able to control cutaneous infection at muzzle and tail sites but failed to clear infection at mucosal sites such as the anogenital tract and the oral cavity. This result demonstrates that immune responses are not equally effective at all anatomical sites. We hypothesize that factors in the local microenvironment may play a role in this differential disease outcome as we observed in NOD/SCID mice, a mouse strain with T, B and NK cell deficiencies. These mice showed a pattern of mucosal infection similar to that in outbred nude mice but, unlike the outbred nude mice, were more resistant to cutaneous infections. Further studies will investigate the role of local host defenses including innate immunity in the control of viral infection at cutaneous sites and the role of innate immune cells in disease clearance.

The finding that MmuPV1 can establish persistent infection in the anogenital tracts of at least two strains of immunocompetent mice and that those infections in the NU/J heterozygotes progress to carcinoma *in situ* over time is important. Lesion classification was based on the degree and extent of dysplasia and the presence or absence of progressive differentiation luminal to the basement membrane. To detect progression of disease in the living animals, we collected vaginal lavages periodically for cytological analysis. This technique is the basis of the Pap smear test used in clinical practice to identify hyperplastic changes in human samples. The most common human cancer associated with papillomavirus is carcinoma of the cervix and most often it is associated with HPV16. The MmuPV1 infections did reach the cervix and we hypothesize that had the animals been maintained longer, these sites, too, would have progressed to carcinoma. We should note that papillomavirus-associated cancers of the human vagina while less common than those of the cervix, do occur^38^.

We have not detected hyperplastic changes in the oral epithelium of the NU/J mice in the current study. We hypothesize that the difference in the disease outcome is due to the genetic background of mouse strains. In our published studies, we identified dysplasia in the oral infections of Hsd Nude mice^22^. In that study, we found hyperplasia and dysplasia in lesions of the circumvallate papillae of the tongues. These back-of-tongue sites are anatomically related to the sites of human papillomavirus-associated oral cancers.

Ever since investigators have been aware of the association between papillomaviruses and human cancers, a suitable mouse model to study the disease and its progression has been sought^12^. Not until 2011 was a papillomavirus that could infect a common laboratory mouse discovered^13^. This virus was identified in a colony of immunocompromised mice and was reported to be strictly cutaneous but our work and that of others has shown both cutaneous and mucosal tropism^30^. The work reported here clearly shows the potential for genital infections with this virus to progress to cancer and to do so in at least one immunocompetent strain of laboratory mouse. This observation paves the way for the establishment of a robust model in which to study disease progression at a site relevant to human infections and in an animal with a competent immune system.

## Materials and Methods

### Viral stock

Infectious virus was isolated from lesions on the tails of mice from our previous study^25^. In brief, the lesions were scraped from the tail with a scalpel blade and homogenized in phosphate-buffered saline (1 × PBS) using a Polytron homogenizer (Brinkman PT10–35) at highest speed for three minutes while chilling in an ice bath. The homogenate was spun at 10,000 rpm and the supernatant was decanted into Eppendorf tubes for storage at −20 °C. For these experiments, the virus was diluted 1:5 in 1 × PBS and 200 μl was passed through a 0.2μm cellulose acetate sterile syringe filter. This was chased by the addition of 200 μl 1 × PBS. The PBS filtrate was added to the filtered virus to give a total of 250 μl sterile virus solution when taking into account loss in the filter. Viral DNA was quantitated by extraction of the DNA from 5 μl of this stock. 1 μl of the DNA extract contains 1.4 × 10^7^ viral genome equivalents^23^. About 1 × 10^8^ viral DNA genome equivalents were used for each infection.

### Animals and viral infections

All mouse work was approved by the Institutional Animal Care and Use Committee of Pennsylvania State University’s College of Medicine (COM) and all methods were performed in accordance with guidelines and regulations. Hsd: NU outbred homozygotes (Foxn1^nu/nu^) and heterozygotes (Foxn1^nu/+^) mice (6–8 weeks) were obtained from Harlan Laboratories (ENVIGO), outbred hairless euthymic SKH1-Elite (Crl: SKH1-Hrhr), inbred C57BL/6 J mice [wild type, homozygotes(Foxn1^nu/nu^), and heterozygotes(Foxn1^nu/+^)], NU/J [outbred homozygotes (Foxn1^nu/nu^) and heterozygotes (Foxn1^nu/+^)] and NOD.CB17-Pkdc^scid^/SzJ (NOD/SCID) were obtained from the Jackson Laboratory (Table 1). All animals were housed (2–3 mice/cage) in sterile cages within sterile filter hoods and were fed sterilized food and water in the COM BL2 animal core facility. Mice were sedated i.p. with 0.1 ml/10 g body weight with ketamine/xylazine mixture (100 mg/10 mg in 10 mls ddH_2_O). For vaginal infection, mice were inoculated subcutaneously with 3 mg Depo-Provera (Pfizer) in 100 µl PBS three days before the viral infection as described previously^23^. Depo was not administered for anal and oral infections. The vaginal and anal tracts were wounded with Doctors’ Brush Picks coated with Conceptrol (Ortho Options, over the counter). Twenty-four hours after wounding, the mice were again anesthetized and challenged with 25 μl (3.5 × 10^8^) and 10 μl (1.4 × 10^8^) of the sterilized viral suspension at the vaginal and anal tracts respectively^24^. For tongue infection, tongues were withdrawn using a sterile forceps and microneedles were used to wound the ventral surface of the tongues^22^. Care was taken to minimize the bleeding. The following day, each animal was again anesthetized. The ventral surface of tongues was again gently abraded and 10 µl of sterile virus (1.4 × 10^8^) was applied to the freshly abraded surfaces. Animals were placed on their backs during recovery to minimize loss of virus from the infection sites. Monitoring was conducted weekly and a photographic log was created for each animal^23^.

### Vaginal, anal, and oral lavage for DNA extraction

The vaginal, anal and oral lavages were conducted using 25 μl of sterile 0.9% NaCl introduced into the vaginal and anal canals with a disposable filter tip. The rinse was gently pipetted in and out of the vaginal canal and stored at −20 °C before being processed. For oral lavage, a swab (Puritan purflock Ultra, puritan diagnostics LLC) soaked in 25 μl of sterile 0.9% NaCl was used. For DNA extraction, the DNeasy kit (QIAGEN) was used according to the instructions of the manufacturer. All DNA samples were eluted into 50 µl EB buffer^23^.

### Mouse CD4/CD8 depletion

Mouse CD4 and CD8 depletions were conducted according to the literature with some modification^17^. Mice were injected with 300 µg of anti-mouse CD4 (Clone GK1.5) and 300 µg of anti-mouse CD8 (clone 2.43 against CD8a) I.P. in 200 µl 1 × PBS at day 1, 2, 3 and challenged with 10 µl (1 × 10^7^) MmuPV1 on day seven. All of the mice were treated with anti-CD4 and anti-CD8 twice weekly until week seven and once weekly after week seven. Two control mice without any treatment were used as positive controls. At week ten after viral infection, blood samples were collected from these animals and examined for the efficiency of T-cell depletion (BD Biosciences, Anti-CD4–APC (clone RM 4–5; BD Pharmingen) and Anti-CD8a-PE and Anti-CD8b-FITC (clone53-5.8; BD Pharmingen) with flow cytometry. Viral DNA was examined from the anal lavage at weeks three and six post-infection. Blood samples were also collected from these animals for anti-MPV antibody detection by standard ELISA.

### Viral DNA copy number analysis

Linearized MmuPV1 genome DNA was used for standard curve determination by SYBR Green Q-PCR analysis (FastStart Universal SYBR Green Master (Rox), Roche). The primer pairs (5′GCCCGA AGACA ACACCG CCACG3′ and 5′CCTCCGCCTC GTCCCCA AATGG 3′) that amplify E2 were used. Viral copy numbers in 1 µl of 50 µl DNA extract from a lavage sample were converted into equivalent DNA load using a formula (1ng viral DNA = 1.2 × 10^8^ copy number, http://cels.uri.edu/gsc/cndna.html). Viral copy number per sample initially provides a simple positive or negative answer and is well adapted to a clinical setting^23^. The Q- PCR reactions were run in a Stratagene Mx Pro-Mx3000 P (Stratagene). Each reaction consisted of 7.5 µl of ultrapure water, 5pmol of each primer, 7.5 µl of SYBR Green-PCR Master Mix (Roche) and 1 μl of DNA template. PCR conditions were: initial denaturation at 95 °C for 10 min, then 40 cycles at 95 °C for 15 s and at 60 °C for 1 min. All samples were tested in at least duplicates. Viral titers were calculated according to the standard curve. In some cases we also calculated the difference in cycle time (Ct) between the 18sRNA gene and viral DNA (ΔCt). Fold change (2^ΔCt^) demonstrates the relative viral DNA load in each sample as described previously^23,39^.

### Antibody detection by ELISA

Mouse sera were collected at the termination of the experiment. Mouse papillomavirus virus-like particles (VLPs) or HPV16 VLPs were used as the antigen for ELISA. Anti-MmuPV1 monoclonal antibody (MPV.B9) and Anti-HPV16 monoclonal antibody (H16.V5) were used as the positive and negative control for the corresponding antigens respectively^23^. The ELISA was conducted as reported previously^40^.

### *In vitro* neutralization assay

A mouse keratinocyte cell line (K38, a generous gift from Dr. Julia Reichelt^41^, University of Newcastle, UK) was seeded at 1.5 × 10^5^ cells per well in DMEM/Ham’s F-12, with 4.5 g/l D-Glucose, 50uM CaCl2, with L-Glutamine and Na-Pyruvate (Cedarlane), in 10% FBS with calcium depleted at 32 °C. 1 µl of viral extract from tail papillomas was incubated with various dilutions of mouse sera (1:50–1:100 dilution) in media for 1hr at 37 °C and added onto K38 cells incubated in 12-well plates at 32 °C for 72 hours. The cells were harvested with TRIzol reagent (Life Technologies). Total RNA was extracted from the infected cells, and infectivity was assessed by measuring viral E1^E4 transcripts with QRT-PCR (E1^E4-forward, 5′-CATTCGAGTC ACTGCTTCTGC-3′; E1^E4-reverse, 5′-GATGCAGGTTTGTCGTTCTCC-3′; E1^E4-probe, 5′-6-carboxyfluorescein (FAM)-TGGAAAACGATAAAGCTCCTCCTC AGCC-6-carboxytetramethylrhodamine (TAMRA)-3′ as previously described with a few modifications ^17^as follows: The Brilliant II Master mix kit (Agilent) was used for the QRT-PCR reactions. The following cycling conditions were applied: 50 °C for 30 minutes (the reverse transcription), 95 °C for 10 minutes, and 40 cycles of 94 °C for 15 seconds and 60 °C for 1 minute. At the end of each amplification cycle, three fluorescence readings were detected. Analysis of the amplification efficiencies was performed using the REST software^42^.

### Immunohistochemistry and *in situ* hybridization analyses of infected tissues

After termination of the experiment, the animals were euthanized and tissues of interest were fixed in 10% buffered formalin as described previously. Hematoxylin and eosin (H &E) analysis, *in situ* hybridization (ISH) and immunohistochemistry (IHC) were conducted as described in previous studies^22,24^. For IHC, a goat group specific antibody (GSA) to a conserved region of L1 (ViroStat #5001), and an in-house anti-MmuPV1 L1 monoclonal antibody (MPV.B9) were used on FFPE sections. Rat anti-mouse LY6.B2 (MCA771GT, Bio-Rad) was used for neutrophil detection.

### Statistical analysis

The data were statistically analyzed with one-way ANOVA analysis for multiple groups in Sigmaplot 12 software. Unpaired student T-test was also used to compare two different groups of animals after viral infections in the studies. Differences were considered to be significant at P < 0.05.

## Electronic supplementary material

Dataset 1
