# Supplementary material for: Mouse papillomavirus infection persists in mucosal tissues of an immunocompetent mouse strain and progresses to cancer
Source: Sci Rep. 2017 Dec 5;7:16932. doi: 10.1038/s41598-017-17089-4 (PMC5717108; doi:10.1038/s41598-017-17089-4)
Supplement: Supplementary file 1 — Dataset 1 [file 41598_2017_17089_MOESM1_ESM.doc]

**Mouse papillomavirus infection persists in mucosal tissues of an immunocompetent mouse strain and progresses to cancer**

Nancy M. Cladel ^1,2^, Lynn R. Budgeon^1,2^, Karla K. Balogh^1,2^, Timothy K. Cooper^3,#^, Sarah A. Brendle^1,2^, Neil D. Christensen^1,2,4^, Todd D. Schell^4^, and Jiafen Hu^1,2,*^

^1^The Jake Gittlen Laboratories for Cancer Research; Pennsylvania State University College of Medicine, Hershey, Pennsylvania, United States of America

^2^ Department of Pathology, Pennsylvania State University College of Medicine, Hershey, Pennsylvania, United States of America

^3^ Department of Comparative Medicine, Pennsylvania State University College of Medicine, Hershey, Pennsylvania, United States of America

^4^ Department of Microbiology and Immunology, Pennsylvania State University College of Medicine, Hershey, Pennsylvania, United States of America

# Current address: Charles River Laboratories – Contractor Supporting: National Institute of Allergy and Infectious Diseases (NIAID) Integrated Research Facility, Division of Clinical Research, 8200 Research Plaza – Fort Detrick, Frederick, MD 21702

**Supplementary Figures**

**Supplementary Figure 1. Viral DNA could be detected in the lower genital tract of infected mice and antibodies against the virus were produced.** Viral DNA was detected in the genital tracts of a subset of the SKH1-Elite (A, N=4) and C57BL/6 (B, N=8) mice at week 2 post-infection but the signals became undetectable by week 4 post-infection. Specific anti-MmuPV1 capsid antibodies were detected in the sera of these animals at week 5 post-infection; C, SKH- Elite and D, C57BL/6. HPV16VLPs were used as the negative control for antibody detection in this study.

**
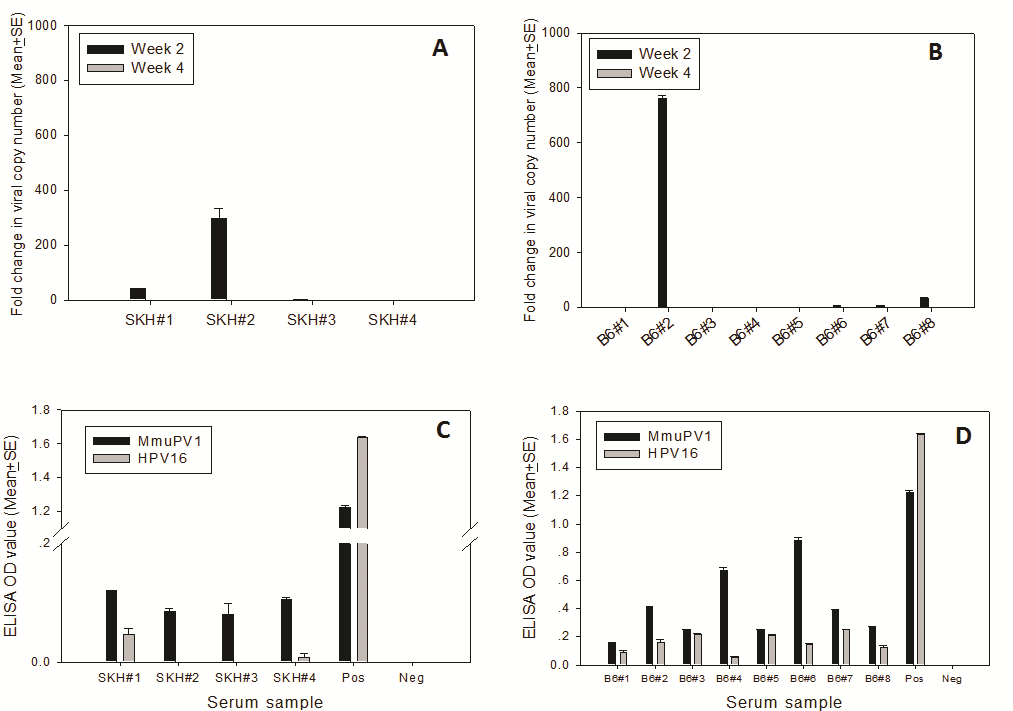
**

**Supplementary Figure 2.Viral DNA could be detected in the oral cavity of C57LB/6 mice and antibodies against the virus were produced.** Eight C57LB/6 mice were infected with MmuPV1 in the oral cavity. Two mice with the highest viral DNA copy numbers from the oral swabs were sacrificed for histological analysis at week 3 post-infection. In the remaining animals, viral DNA was detected at week 3 post-infection and became undetectable after week 4 (A). These six animals were sacrificed at week 7 post-infection. Serum samples were harvested from all animals for antibody detection. No viral DNA was detected at the infected sites by *in situ* hybridization analysis. However, all eight infected mice generated detectable antibodies against mouse papillomavirus (B).

**
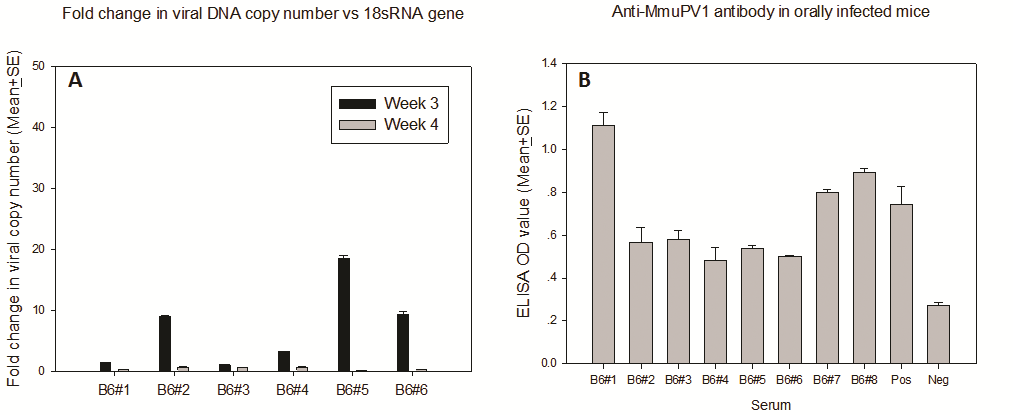
**

**Supplementary Figure 3.CD4 and CD8 depletion in C57LB/6 mice resulted in increased copy numbers of viral DNA in the anal canal.** Three C57LB/6 mice (2-4L, 2-4R and 2-4No) were infected at the anal canal as described previously. CD4 and CD8 T cells were depleted with anti-mouse CD4 (Clone GK1.5) and anti-mouse CD8 (clone 2.43 against CD8a) at day 1, 2, 3 before viral infection on day 7. All the mice were treated with anti-CD4 and anti-CD8 twice weekly until week 7 and once weekly after week 7. Two control mice without any treatment were used as negative controls (6-4L and 6-4R). At the termination of the experiment, CD8 T cell populations (A) were significantly reduced (C, P<0.05 vs. the control, unpaired Student T-test) but change in CD4 cell populations (B) was not significant in the spleen (C, P>0.05 vs. the control, unpaired Student T-test). Viral DNA fold changes of individual animals were shown in D. Significantly more viral DNA copy numbers were detected in the depleted mice when compared with that of the control group at week five post-infection (E, P<0.05, unpaired Student T-test).

**
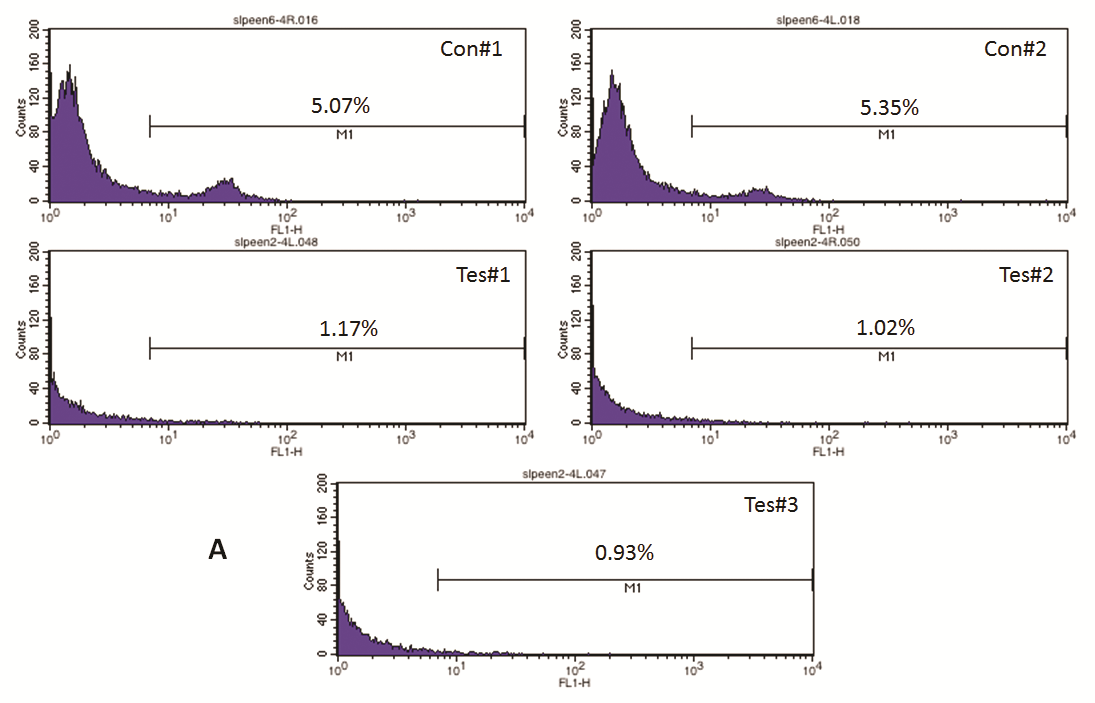

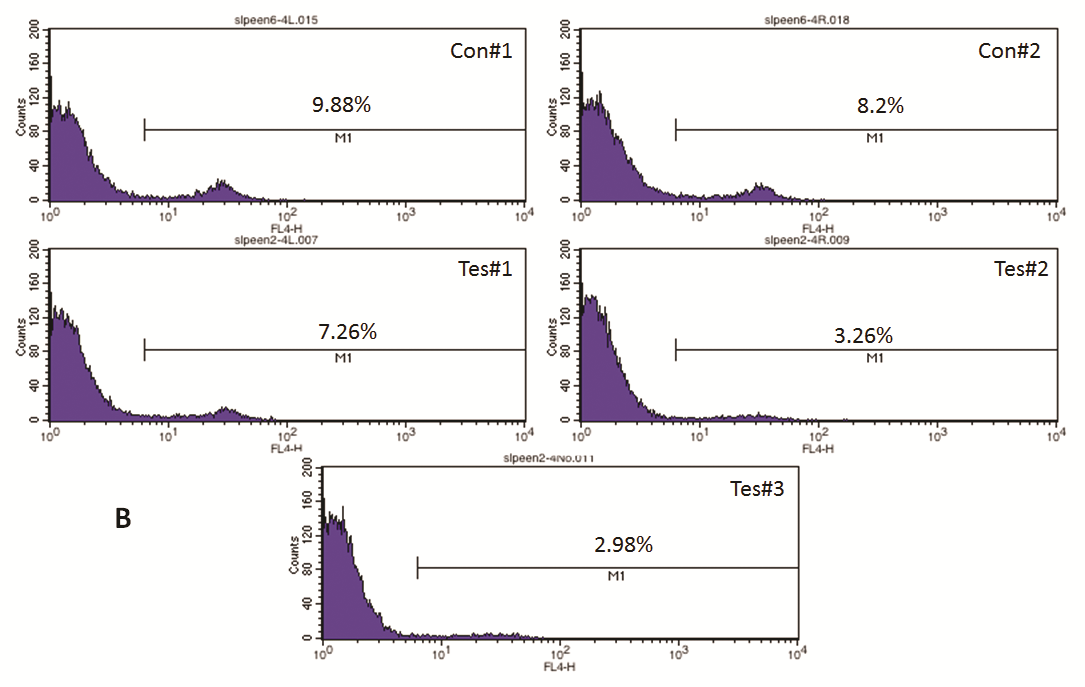

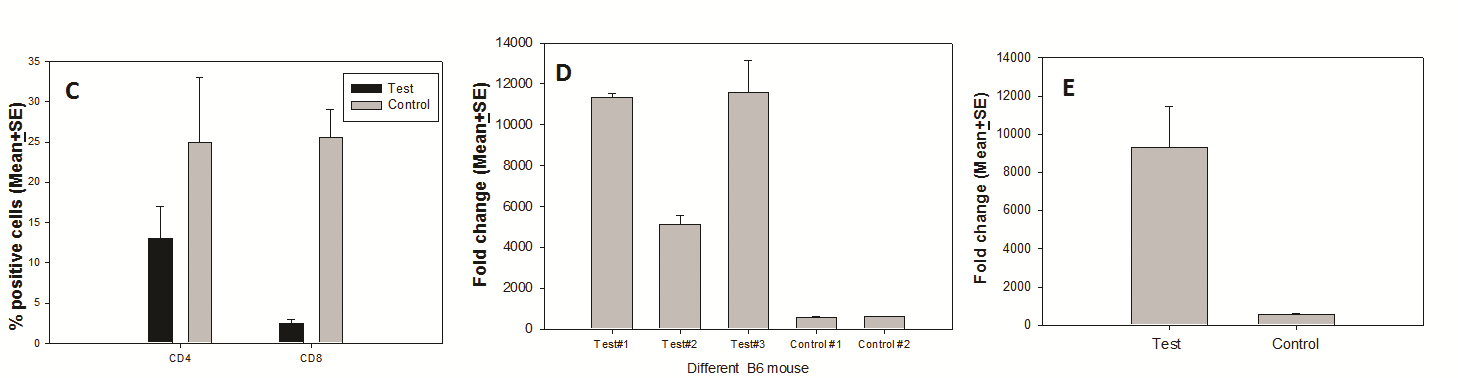
**

**Supplementary Figure 4. Sera harvested from MmuPV1-infected heterozygous NU/J mice exhibited strong antibody titers against MmuPV1 and are neutralizing the virus**. All heterozygotes generated high-titers of anti-MmuPV1 antibodies while one of the three examined homozygous mice also showed strong positivity (A). All the serum samples were neutralizing (B).

**
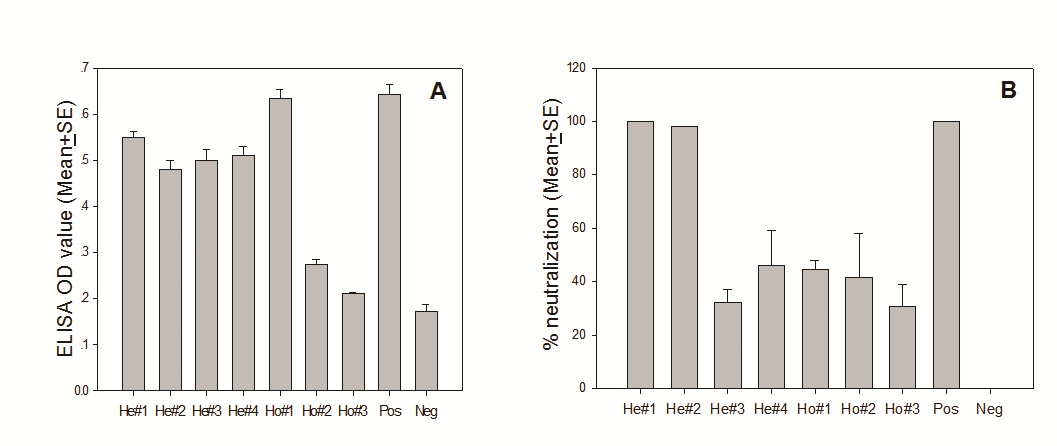
**

**Supplementary Figure 5.** **Viral infections in IFNα/βR knockout mice prolonged when compared with those in the wild type mice.** Viral DNA can be detected in lavage samples of the anal tract of one male (1-7) and two females (2-7L and 2-7R) (A) as well as of the lower genital tract of 2-7L and 2-7R (B) of Ifnar-/- mice. In contrast, viral infections were cleared in the wild type B6 mice before week five post infection (supplementary Figure 1 and 3).

**
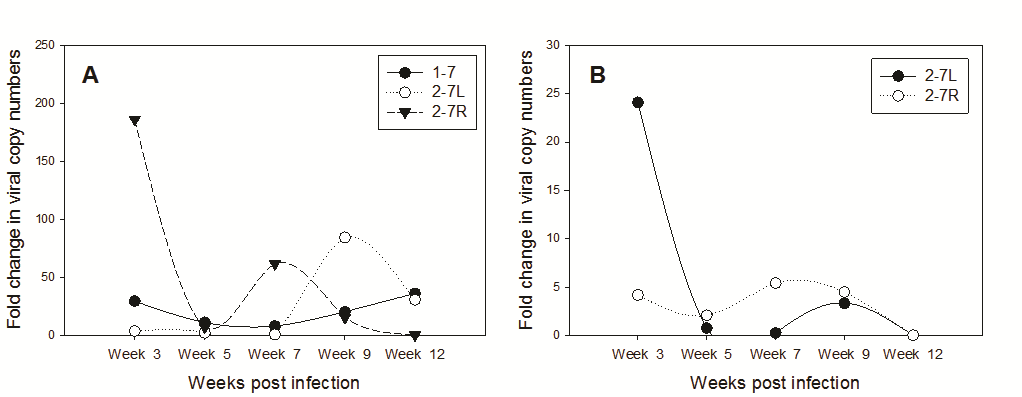
**
